# New insights into how seeds are made

**DOI:** 10.3389/fpls.2015.00196

**Published:** 2015-03-26

**Authors:** Paolo A. Sabelli, Brian A. Larkins

**Affiliations:** ^1^Department of Plant Sciences, University of ArizonaTucson, AZ, USA; ^2^Department of Agronomy and Horticulture, University of NebraskaLincoln, NE, USA

**Keywords:** seed development, seed metabolism, seed genetics, seed epigenetics, seed evolution, seed physiology, seed genomics, seed germination

It is difficult to overstate the importance of seeds for the evolutionary success of the spermatophytes and the development of human cultures and their survival. For hundreds of millions of years since their appearance in the Devonian period, seed plants have colonized many environments, thanks in part to several adaptations, including seed formation. Seeds can spread over large areas over long time spans, while also protecting the embryo from pathogens and adverse environmental conditions. Seeds vary greatly in shape and structure, but they typically possess three major components: the sporophyte (*i.e*., the embryo), a storage metabolite compartment (*i.e*., cotyledons, endosperm or perisperm) enabling the early growth of the seedling before it is autotrophic, and a protective coat. Since agriculture began around 10,000 years ago, certain seed plants have been domesticated and methodically bred to provide food and raw materials. Today, vast swaths of land are planted to a few grain crops that have proven highly nutritious and productive. In view of the exploding (and generally more affluent) human population, which is projected to reach nine billion by mid-century, crop genetic erosion, constrained fossil fuel availability, and global climate change put enormous pressure on agriculture. The continuous genetic improvement of seed plants has played a key role in sustaining the human population for thousand of years, but additional improvements are necessary in the future. A clear understanding of the biological processes controlling seed development, quality, and yield is required to meet the challenges facing agriculture.

Our comprehension of seed development has progressed considerably, thanks to model plant systems, development of high through-put techniques, and increased understanding of genetic and biochemical pathways. The goal of this Research Topic on Advances in Seed Biology is to update key aspects of seed development, evolution and physiology.

The first step in seed formation is a double fertilization event, which involves the egg cell and central cell in the female gametophyte each fusing with a sperm cell. This process is tightly regulated, and Bleckmann et al. (2014) describe the complex signaling pathways that control pollen tube growth, communication with female flower structures, and gametes interaction. The fertilized egg and central cell go on to form the embryo and the endosperm, respectively, by multiplying and expanding through several variant cell cycles, such as mitotic cell proliferation, acytokinetic mitosis, and endoreduplication. Dante et al. (2014) and Sabelli et al. (2014) describe core cell cycle factors that play important roles in the regulation of the cell division cycle during seed development and its coordination with cell differentiation and maturation.

Seed development and the success of sexual plant reproduction are affected by the ploidies of the parental gametes and by epigenetic mechanisms causing parent-of-origin-dependent gene expression (*i.e*., genomic imprinting). These phenomena are commonly interpreted according to the parental-conflict hypothesis, but a reassessment may be in order. In his perspective on maize, Birchler (2014) focuses on crosses between parents with abnormal genome ratios, which generally result in endosperm failure and seed abortion. He proposes this interploidy barrier can be explained primarily by genome dosage interaction between the female gametophyte and the triploid primary endosperm nucleus. Furthermore, based on information derived from large transcriptomic studies, Bai and Settles (2015) observe that genomic imprinting, although widespread among angiosperms, is neither highly conserved with respect to the nature and patterns of genes or genome fractions involved, nor does it appear to be necessary. Thus, these authors propose imprinting may represent an evolutionary strategy that, through specific epigenetic regulation early in seed development, allows rapid evolution and neofunctionalization of new alleles without necessarily compromising fitness of the adult sporophyte.

Becker et al. (2014) present an overview of transcriptome-based analyses for unraveling gene networks and pathways controlling seed development, which are especially valuable when coupled with the ability to isolate relatively homogeneous cell populations.

Li and Li (2014) review regulation of protein degradation by the 26S proteasome as an emerging critical mechanism by which seed size and organ growth are controlled. Burton and Fincher (2014) take an evolutionary perspective to compare composition of cell walls in cereal grains, which are particularly rich in (1,3;1,4)-β-glucans, to those of other monocots and dicots.

A number of articles focus on seed metabolism. Galili et al. (2014) discuss how seed metabolism and energy status are impacted by low levels of oxygen, photosynthetic activity, and effects on the Asp-family of amino acids. Wu and Messing (2014) discuss how different proteins and sulfur are dynamically balanced in developing maize seeds and describe the outcomes of experiments aimed at shifting the seed proteome to increase seed nutritional value. Herman (2014) analyses the interplay between protein content and composition in the soybean proteome and the critical roles played both by genetic and physiological factors. Arcalis et al. (2014) review pathways affecting protein trafficking and storage organelle development in cereal seeds and discuss their significance with regard to our ability to produce recombinant proteins through transgenic approaches. Mainieri et al. (2014) show accumulation of the maize 27-kD γ-zein in protein bodies depends on several cysteine residues in its *N*-terminal domain, which results in insoluble endoplasmic reticulum (ER)-localized prolamin accretions. Holding (2014) provides an update on cereal storage protein accumulation, particularly in maize, and on past and present strategies to study their function in developing endosperm.

Ravel et al. (2014) present a detailed analysis of promoter regions in wheat seed storage protein alleles and identify conserved and unique motifs. Burrieza et al. (2014) draw several important analogies between quinoa and grass seed structures, suggesting these species underwent convergent evolution with regard to seed structure and the function of specific compartments. Domínguez and Cejudo (2014) discuss the regulation of programmed cell death and how it affects different tissues during cereal seed development and germination. Radchuk and Borisjuk (2014) assess roles of the seed coat in protection, transducing environmental signals to inner seed compartments and influencing seed size. Smýkal et al. (2014) provide a detailed discussion of the role of the testa in controlling legume seed germination through its physical and chemical properties. And last but not least, Nonogaki (2014) provides a critical and stimulating review of the mechanisms involved in seed dormancy and germination, highlighting the emergence of new paradigms integrating genetics, the action of hormones and epigenetic modifications of chromatin conformation and gene activity.

## Conflict of interest statement

The authors declare that the research was conducted in the absence of any commercial or financial relationships that could be construed as a potential conflict of interest.
